# Effect of vitamins C or E supplementation to Tris diluent on the semen quality of Awassi rams preserved at 5 ˚C

**Published:** 2013

**Authors:** Osama Ibrahim Azawi, Elias Khudhur Hussein

**Affiliations:** *Department of Surgery and Theriogenology, College of Veterinary Medicine, University of Mosul, Mosul, Iraq.*

**Keywords:** Awassi ram, Semen, Tris, Vitamin C, Vitamin E

## Abstract

The present study was aimed to test the efficacy of adding vitamins C or E to Tris-fructose-egg yolk diluent to increase Awassi ram sperm storage period at 5 ˚C. Semen samples from six mature Awassi rams were used in this study. The semen samples were diluted by Tris-glucose-egg yolk. Diluted semen sample was divided into three parts. The first part was added with 0.9 mg mL^-1 ^vitamin C, the second part was added with 1 mg mL^-1 ^vitamin E and the third part was considered as a control without any addition. The diluted semen samples were cooled gradually and preserved at 5 ˚C for five days. Sperms in cooled diluted semen samples were examined for motility, vitality, abnormalities and acrosomal defects every 24 hr for five days. Results of the present study showed an increase in the viability of spermatozoa diluted in the Tris diluent containing vitamins C or E stored at 5 ˚C for 120 hr compared with the control group. There were significant (*p* < 0.05) effects of vitamins C and E addition to semen diluents on sperm motility as well as the sperm viability in different times of preservation at 5 ˚C. Significant (*p* < 0.05) higher sperm abnormalities and acrosomal defects values (37.6 ± 1.3% and 71.5 ± 1.1%, respectively) were found after 120 hr incubation in Tris free vitamin C (Control) at 5 ˚C compared with those of containing vitamin C (18.8 ± 1.8% and 52.8 ± 4.3%, respectively). From the results of the present study, it could be concluded, that the addition of antioxidants such as vitamins C and vitamin E to semen preservation media could improve longevity and quality of cooled sperm in Awassi ram semen.

## Introduction

Sperm cells have a high content of unsaturated fatty acids in their membranes, while lacking a significant cytoplasmic component containing antioxidants. Therefore, sperm cells are very susceptible to lipid peroxidation by free radicals such as hydrogen peroxide, superoxide anion, and hydroxyl radical, which could later lead to the structural damage of sperm membranes during the aerobic storage of sperm.^1^ The cooling and aerobic preservation processes produce physical and chemical stress on the sperm membrane which sequentially reduces sperm viability and fertilizing ability. Cold shock of sperm cells during the cooling process is associated with oxidative stress induced by free radicals.^2^^,^^3^ Free radicals seek stability by “stealing” electrons from nucleic acids, lipids, and proteins leading to the damage of cells.^4^ Free radicals are mostly eliminated by antioxidant systems. Antioxidants play an important role in scavenging free radicals which otherwise may cause lipid peroxidation of sperm plasma membranes.^5^

Vitamin C (ascorbic acid or ascorbate) and vitamin E (tocopherol) are non-enzymatic antioxidants.^6^ The addition of anti-oxidants is well known method to improve viability and motility of liquid storage or cryopreserved ram sperm cells.^7^ The most important antioxidants in seminal fluid seem to be vitamins C and E.^8^^,^^9^ The concentration of vitamin C in seminal plasma is 10 times greater than in blood plasma (364 vs. 40 μmol L^-1^).^10^ Hughes *et al*. found that *in vitro *treatment of sperm with antioxidants (300 and 600 μM ascorbic acid with 3 and 60 μM alpha-tocopherol) reduced the magnitude of DNA damage.^11^ Vitamin E inhibits lipid peroxidation in membranes by scavenging peroxyl (ROO^-^) and alkoxyl (RO^-^) radicals.^12^ However, the ability of α-tocopherol to maintain a steady-state rate of peroxyl radical reduction in the plasma membrane depends on the recycling of α-tocopherol by external reducing agents such as ascorbate or thiols.^13^^,^^14^ Although much work has been done by the addition of antioxidants to cattle semen, information on their use in sheep especially Iraqi Awassi rams are scares. Therefore, the present study was undertaken to test the efficacy of adding vitamins C or E to Tris-fructose-egg yolk diluent on sperm quality of the Awassi ram semen stored at 5 ˚C.

## Materials and Methods

**Animals and semen collection. **Semen samples from six mature Awassi rams (2-3 years of age) were used in this study. The rams were fed, housed and lit, conventionally. The study was carried out from September 2010 to June 2011. Animals were housed at the Animal Research and Practice Farm of College of Veterinary Medicine, University of Mosul (36^o^20' N, 43^o^ 8' E), Iraq. All the rams were apparently in good health. They were maintained in equal nutritional and managerial condition throughout the period of the study. Throughout the experimental period, the animals were kept in open front barrens, were fed individually with concentrated mixture (Containing 65% barley, 33% bran, and 2% minerals and salt mixed in the farm) of 1 kg per ram per day, and water was given *ad libitum*. A total number of 60 ejaculates were collected from the rams using an artificial vagina once a week. For collecting ejaculates, rams were penned with ewes in estrus, in the presence of a handler with an artificial vagina. Ejaculates were evaluated and included in this study if the following criteria were met: volume of 0.5-2 mL; sperm concentration of 1-2 × 10^9^ sperm per mL; the progressive motile sperms percentage higher than 70% and abnormal sperm of less than 10%.

**Semen analysis. **The volume of each ejaculate was recorded and sperm concentration was determined using semen diluted with 3% NaCl, the diluted semen was placed on a hemocytometer with the sperm counted in five squares of one chamber. Sperm motility was identified as those sperm cells that demonstrated progressive motility. Sperm motility was scored from zero to 100% by a qualified and experienced investigator. Semen was placed on a heated glass slide, and scoring was performed at microscopic magnification of 200×. Each sample was evaluated twice. The mean value was used for data analysis. Assessment of abnormal and normal spermatozoa was performed using an eosin–nigrosin staining method^15^*. *A fast green stain was used for the estimation of spermatozoa with abnormal acrosomes.^16^

**Dilution of semen and addition of vitamins C and E. **Semen samples were diluted by Tris-fructose-egg yolk (Hydroxy methyl amino methane-Tris 3.6 g, glucose 0.5 g, citric acid 1.7 g, streptomycin sulphate 100 mg, crystalline penicillin 100000 IU, double distilled water 85 mL and egg yolk 15 mL). Semen quality was re-evaluated to ensure that the dilution had not affected the semen quality. Diluted semen sample was divided into three parts. The first part was added with 0.9 mg mL^-1 ^vitamin C, the second part was added with 1 mg mL^-1 ^vitamin E and the third part was considered as a control without any addition. The diluted semen samples were cooled gradually and preserved at 5 ˚C for five days. Cooled diluted semen samples were examined for individual sperm motility, vitality, abnormalities and acrosomal defects every 24 hr for five days. 

**Statistical analysis. **Data for the percentages of motility, live sperm, abnormal sperm and acrosomal defects were arcsine transformed. Statistical analyses were performed with Sigma Stat (Version 2.0, Jandel scientific software, Richmond, CA, USA). Data were expressed as mean ± SEM. Variance of homogeneity of samples was examined by Levene's test. The differences between means of the same parameter were tested by the ANOVA and LSD tests. 

## Results

**Fig. 1:**
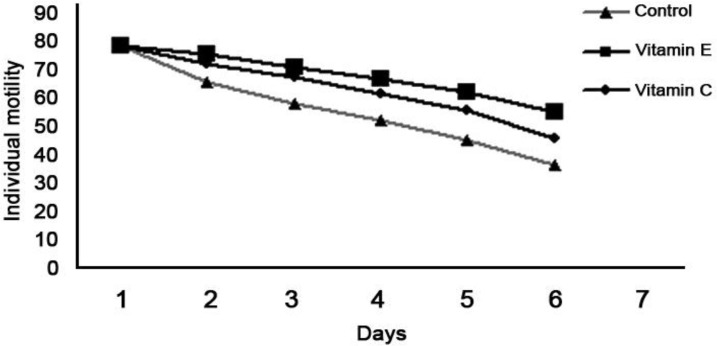
Percent of progressive individual motility of Awassi ram semen diluted with Tris diluent containing vitamin C, E and control groups stored at 5 ˚C for 120 hr.

In this experiment no significant differences (*p* > 0.05) were found between individual rams in the evaluated parameters. Ram effect was eliminated from the model. Results of the present study showed an increased viability of spermatozoa diluted in Tris diluent containing vitamins C or E stored at 5 ˚C for 120 hr compared with the control group. The effect of adding vitamins C and E to Awassi ram semen diluted with Tris on sperm characteristics following the preservation at 5 ˚C for 120 hr are presented in Table 1. Spermatozoa motility declined gradually in vitamins C and E Tris containing diluents when preserved at 5 ˚C from 0 to 120 hr to 54.7 ± 1.0% and 45.5 ± 1.4%, respectively, compared to Tris diluent without vitamins which was declined markedly to 35.9 ± 1.9% (Fig. 1). There were significant major effects of vitamins C and E addition to semen diluents on sperm motility as well as sperm viability in different times of preservation at 5 ˚C (*p* < 0.05). A significant (*p* < 0.05) higher sperm abnormalities and acrosomal defects values (37.6 ± 1.3% and 71.5 ± 1.1%, respectively) were found after 120 hr incubation in vitamin C free Tris (Control) at 5 ˚C compared with those obtained in Tris diluent containing vitamin C (18.8 ± 1.8% and 52.8 ± 4.3%, respectively). Adding vitamin C to Tris diluent showed more protective effect than vitamin E. Moreover, addition of vitamin C resulted in a statistically significant decrease (*p < *0*.*05) in sperm abnormalities and acrosomal defects of Awassi ram semen after 120 hr of incubation at 5 ˚C. 

**Table 1:** Values of individual motility, live sperms, abnormal sperm and abnormal acrosomes of Awassi ram semen diluted and stored at 5 ˚C for 120 hr with Tris diluent containing vitamins C and E in contrast to the control group. Data are presented as mean ± SEM.

**Groups**	**Individual motility ** **(%)**	**Live sperms ** **(%)**	**Abnormal sperms ** **(%)**	**Abnormal acrosomes ** **(%)**
**Tris with vitamin C**	54.7 ± 1.1^a^	59.7 ± 1.3^a^	18.8 ± 1.8^a^	52.8 ± 4.3^a^
**Tris with vitamin E**	45.5 ± 1.4^a^	50.5 ± 1.5^a^	35.3 ± 1.9^b^	62.7 ± 2.9^b^
**Control**	35.9 ± 1.9^b^	40.9 ± 1.8^b^	37.6 ± 1.3^b^	71.5 ± 1.1^b^

Mean values for each parameter in the same row, with different superscript (a, b) are significantly different (*p* < 0.05).

## Discussion

This study is the first report on the effects of adding vitamins C or E to Tris-fructose-egg yolk diluent for sperm storage at 5 ˚C of Iraqi Awassi rams. Free radicals are mostly eliminated by antioxidant systems. Thuwanuta *et al*. found an improvement of ram sperm after the addition of antioxidants (vitamins C and E) to semen diluents. The results of the present study showed that Awassi ram semen diluted with Tris diluent containing vitamin C or E could improve sperm motility and viability. The addition of vitamin C not only improved viability but it also protected acrosome and membrane integrity. Our results showed that sperm motility and viability of Awassi ram semen diluted with Tris containing vitamin C could be maintained and improved for 120 hr of preservation at 5 ˚C. These results were in agreement with Hughes^11^ of human semen; Maia^13^^, ^^14^ of ram semen and Singh^17^ in buffalo semen. The beneficial effect of adding vitamin C to diluted ram semen was in accordance with the results obtained by Asghari.^18^ Previous studies in humans showed that ascorbate in the range of 0.02-0.6 mM adversely affected sperm motility.^19^ Higher concentrations of vitamin C (2.5 mM) proved harmful to sperm motility in frozen-thawed bull semen.^20^ Vitamin C represents the major water-soluble antioxidant in blood plasma and seminal plasma.^10^ Most animals can synthesize ascorbic acid from glucose via the glucuronic acid pathway. Ascorbic acid is required as a cofactor for at least eight enzymes^21^ and can also act as an antioxidant by reacting with free radicals (e.g. O_2_^-^, OH^-^). However, in the presence of transition metal ions (e.g. Fe^3+^, Cu^2+^) high concentrations of ascorbic acid can act as a pro-oxidant by donating an electron that reduces such ions to forms that, in turn, can react with oxygen molecules to form oxygen radicals. Beneficial effect of supplementation sturgeon sperm with ascorbic acid was demonstrated by Mirzoyan^22^. These authors detected an increase in the motility rate of Russian sturgeon sperm added with 10 mM ascorbic acid. This favorable effect of adding vitamin C to diluted semen seems to relate to a reduction in DNA damage of spermatozoa.^23^ Vitamin C can protect membrane integrity of sperm cells from heat shock during dilution-cooling-storage process of spermatozoa.^10^ A probable improvement in semen quality by addition of vitamin C in ram semen diluent is more likely related to an inhibition of lipid peroxidation of the sperm plasma membrane as was revealed by Barati.^24^ The beneficial effect of vitamin C or vitamin E in improving fertilization rate as found by Hsu,^25^ was possibly due to a reduction in lipid peroxidation of ram sperm.^14^ Vitamin E can inhibit lipid peroxidation reaction in the membrane by eliminating peroxyl (ROO^-^), alkoxyl (RO^-^), and other lipid-derived radicals.^26^ Askasi reported vitamin E was more efficacious than vitamin C in improving post-thaw motility of human spermatozoa.^27^

However, the present study showed vitamin C was more efficient in protecting ram spermatozoa viability and acro-somal integrity. This could be due to the vitamin C capability of neutralizing H_2_O_2 _produced in a hydrophilic environment. It could be concluded that the addition of antioxidants such as vitamins C and E to the preservation media could improve longevity and quality of cooled sperm in Awassi ram semen. 
